# The New Journalism

**Published:** 1887-09-03

**Authors:** 


### THE NEW JOURNALISM.

WHAT is the physiological condition of Mr. Stead's brain, of the *Pall Mall Gazette?* Are the vaso-motors of the hemispheres in working order, or do they sometimes fail of their duty, and give Mr. Stead a sleepless night? And does his want of sleep account for his occasional literary excesses? Those who claim to be teachers and censors should spare a modicum of consideration for their own physical and mental condition. A teacher who is much listened to, and whose school is large, has a degree of responsibility strictly corresponding to the number and attentiveness of his pupils. He cannot teach as he ought if his brain be not healthy, and especially if his prelections be on moral subjects. Enthusiasm is not wisdom: good intentions do not always give expression to sound principles. Great is honest enthusiasm for good; but great also is sense. A man may be a hero and a saint, and at the same time not very far removed from a fool. In a full man, the necessary complement of zeal and goodness is sense, and yet more sense. When a man is zealous, courageous, strong, and wise, what may he not accomplish in twenty years?

Mr. Stead is a typical representative of what is called the "New Journalism." The journalism which is considered by many people so very peculiar is only "new" in the sense that it is new to this generation. There have been journals before the present decade which have made a point of telling the truth as plainly and forcibly as possible. Some of them have even done what they now charge Mr. Stead with doing, and "shrieked" the truth. There is no reason why the truth should not be shrieked if the occasion require it. A loud shriek is sometimes of great service to a person, as for example if a mad dog be tearing after him, and he does not see it. There may be more dangerous things than mad dogs. A mad man with a supply of dynamite in the cellars of the House of Lords might be critically dangerous; and if Mr. Stead knew about such a man, and were to shriek, even loudly and indecorously, at the door of the Painted Chamber, it is probable their lordships would forgive him. It would be worth a two-guinea fee to see the dignified haste with which they would all get themselves outside.

But a mad man is sometimes less dangerous than a bad man. Given a downright scoundrel; base, selfish, sensual; as base, vulgar, and detestable in his baseness as even the devil could wish him to be: given such a man, every person who has been taught the elements of decency and morality will cry "stop" if he sees him in full career. Anyone who finds out that there is such a man; that he has done such things that if they were said to have been done by an Indian prince or African savage to a woman, the listener would long to hack the savage to death with a sword; any man finding that these things have been done by an educated white man to an educated and virtuous white woman, and that after doing them the educated white man was anxious to leave his victim and his own child to perish of hunger if possible—any man finding out these things would be in an angry mood, and would take some trouble to get the villain punished. The commonest day-labourer, or tailor even, being a man, a creature with intelligence and conscience, and some little sympathy—such a common man of the herd and rabble would no doubt feel his tailor's blood rushing through his brain and fingers, and would seize the first weapon within reach to give the scoundrel his deserts. But much more would a man of academic education, an admirer of the classical examples of piety and nobility; of the soft and tender spirit of Christianity; a man familiar with Wordsworth and Tennyson; a man perhaps who had gentle sisters and playful innocent daughters of his own—such a man, hearing of such a villain, and knowing he was persisting in his villainy, even to the death, if possible, of her whom he had vowed before God and men to love—would not such a man, if he could, call down fire from heaven like Elijah, to burn up and destroy the monster?

Was that what the editors of the London newspapers did when they knew of the facts of the Langworthy case? Was it what mere humanity, or at least educated sympathy, would have done? It surely was! What then can be said of the London editors, if there were any, who, knowing of the case, passed it by in silence? Let them say what they can to their own manhood, to their own consciences! If the editing of a newspaper destroys a man's plain and simple instincts of right and duty, then there is one person at least who would be pleased if no single newspaper were ever published from this day forward. Nothing can repay a man or a nation for the loss of that which constitutes him the intelligent and conscious sympathiser with the just and perfect God.

Mr. Stead, whatever be his defects of judgment, took up the case of Mrs. Langworthy. However he may spend his life in future; whatever elevation he may attain, or to whatever depths he may even sink; so long as memory lasts he will have the consciousness that he took one poor distressed woman by the hand when she was in the "horrible pit and in the miry clay," and that he never ceased until he had "set her feet upon a rock," and "put a new song into her mouth." Wherever this story shall be told in the whole earth there will the teller and the hearer have a better opinion of mankind. The salt of Mr. Stead's good deed will be more than sufficient to sweeten the moral atmosphere made unwholesome by the unworthy silence of other men whose opportunities were equal to his.

Some people with great names have sullied them for ever. These things can never be forgotten. Whoever allows himself, for money or other consideration, to help the strong and unscrupulous to trample upon and destroy the helpless—what kind of man is he at heart? When Sir Charles Russell spoke at the Memorial Hall the other evening in favour of what he considered a down-trodden nationality, did no voice whisper anything in his heart about the "alleged child"? There were among his hearers some who used to be his admirers. Not a word that he said carried any conviction to their minds. The scene in the law-court was present to them; the paid advocate, knowing that the woman whose cause he opposed was doubly widowed and cast out; knowing that the success of his advocacy meant her ruin and the degradation of her innocent and worse than fatherless child; knowing this—used all the energy of his intellect and all the resources of his art to bring about that ruin and degradation. That scene—present to the minds of some of his hearers—made them turn away in loathing and disgust. Every rounded period raised but one echo in their ears, and that echo spoke the words, never to be forgotten—"The alleged child."

It is a pity, a most grievous pity, that for such a mean and miserable consideration as gold a man of the most distinguished ability and of the highest cultivation will use his utmost powers to persuade a jury to believe that which is a lie. No man is so black but may have some white spots: no cause so bad but may have some extenuating circumstances. Let the white spots be shown; let the extenuating circumstances be pointed out; let the pity of the jury and the clemency of the judge be invoked! These are all right and proper to be done. But in the name of man and in the name of God let not the wicked man triumph because he has wealth, whilst the defenceless woman's cause is lost because she is poor! We cannot, must not, and will not have law degraded and justice made a mockery because men of law have ceased to believe in the simplest elements of right and wrong.

The new journalism, as represented by Mr. Stead, may have many and conspicuous faults. That his judgments should be more influenced by cool reason and less by mere enthusiasm is in the highest degree to be wished for. But when he has in hand a case like that of Mrs. Langworthy and her child, every man and woman who has ordinary virtue, and the least measure of Christian belief, will say to him with the deepest earnestness of conviction—Your work is good: do it with all your might.

